# Morphological and Molecular Characterization of a New Myxozoan, *Myxobolus grassi* sp. nov. (Myxosporea), Infecting the Grass Carp, *Ctenopharyngodon idella* in the Gomti River, India

**DOI:** 10.3390/pathogens11030303

**Published:** 2022-02-28

**Authors:** Naireen Fariya, Harpreet Kaur, Mahender Singh, Rehana Abidi, Mansour El-Matbouli, Gokhlesh Kumar

**Affiliations:** 1 Parasitology Laboratory, Fish Health Management Division, National Bureau of Fish Genetic Resources, Lucknow 226002, India; n_fariya@yahoo.com (N.F.); abidinbfgr@gmail.com (R.A.); 2 Department of Zoology, Panjab University, Chandigarh 160014, India; harpreetbimbra@gmail.com; 3 DNA Barcoding Laboratory, Molecular Biology & Biotechnology Division, National Bureau of Fish Genetic Resources, Lucknow 226002, India; dr.mahendersingh29@gmail.com; 4 Clinical Division of Fish Medicine, University of Veterinary Medicine Vienna, Veterinärplatz 1, 1210 Vienna, Austria; mansour.el-matbouli@vetmeduni.ac.at

**Keywords:** grass carp, myxozoan, new myxozoan species, *Myxobolus grassi*

## Abstract

Myxosporeans are well-known parasites infecting food fishes in fresh and marine water around the globe. Grass carp *(Ctenopharyngodon idella*), a freshwater food fish commonly cultured in India with has significant economic importance. Herein, the study focuses on the description of a new myxosporean species, *Myxobolus grassi* sp. nov. from the gills as primary site and liver as secondary site of infection in grass carp. Both organs (gill and liver) were infected concurrently in the host and the prevalence of grass carp infection was 4.05% in gill filaments and liver, respectively. Identification of species was based on the morphological and morphometric features of the myxospore as well as 18S rDNA sequence data. A smear from gill and liver exhibited hundreds of morphologically similar myxospores. BLAST search revealed 98% sequence similarity and 0.03 genetic distance with *M. catlae* (KM029967) infecting gill lamellae of mrigal carp (*Cirrhinus cirrhosus*) from India and 98–84% sequence similarity with other myxobolids in India, China, Japan, Malaysia, Turkey and Hungary. Phylogenetically, it clustered with other myxobolids infecting gills and related organs (i.e., vital organ) of Indian cyprinid carp species. On the basis of myxospore morphology and 18S sequence, we propose *M. grassi* sp. nov.

## 1. Introduction

Fishes are considered as a healthy source of animal protein, providing essential nutrients to the human diet. Grass carp (*Ctenopharyngodon idella*) is a cyprinid food fish, having a major contribution in freshwater aquaculture globally. It grows quickly with minimal input cost. The fisheries sector can contribute substantially to the elimination of food insecurity and malnutrition in a responsive way [1]. Traditionally, fish culture has been associated with livelihood and income in India, thereby contributing to the economy. Parasitic infections have always received special attention in food fish and ornamental fishes. Moreover, *C. idella* is easily prone to diseases, which affects the health of the fish [2]. Poor management or lack of awareness on better management practices in fish culture might manifest the risk of extensive harmful parasites. Myxozoans are economically important groups of microscopic parasites among freshwater fishes comprising more than 2000 known species [3,4]. The genus *Myxobolus* (Myxobolidae) encompasses largest number of species worldwide [5]. A synopsis of 744 and 112 nominal *Myxobolus* species was compiled by Eiras et al. [6,7] respectively. In addition, Kaur and Singh [8] gave a synopsis of 131 species of *Myxobolus* reported from India. As demonstrated by Kaur [9] new myxozoans are emerging and threatening the development of pisciculture causing production losses. Hitherto, globally many myxozoan infections were recorded in carps and cyprinids [6,7] while several studies on prevalence of myxosporidiasis and novel myxozoan species with negative impact reported in grass carp [8,10,11]. With this view, the parasitic investigation was performed for grass carp, an economically significant freshwater food fish commonly cultured in India. The present study reports morphological identification and molecular characterization based on 18S rDNA of *M. grassi* sp. nov. infecting the gill filaments and liver of grass carp. 

## 2. Results

Taxonomic summary of *Myxobolus grassi* sp. nov.
(Cnidaria: Myxozoa: Myxosporea: Bivalvulida: Myxobolidae: Myxobolus)
Host: *Ctenopharyngodon idella* (Grass carp) (Valenciennes, 1844)
Locality: Gomti river (Kaisar Bagh fish market), Lucknow, Uttar Pradesh, India
Type specimen: Holotype with the slide no. M01–05/SN/GC/10.2.2018 was deposited in the museum of the Department of Zoology, Punjab University, Chandigarh, India
Paratypes: Gills fixed in 4% formalin with catalogue no. M/G/GC/10.2.2018 as paratype has been deposited with Dr. Harpreet Kaur, Department of Zoology, Punjab University, Chandigarh, India
Site of infection: Gill filaments (Vascular epithelium) and liver
Prevalence: 4.05%
Etymology: The present species is termed as *M. grassi* sp. nov. after the common name of the fish host i.e., grass carp

### 2.1. Morphological Description

**Figure 1:**
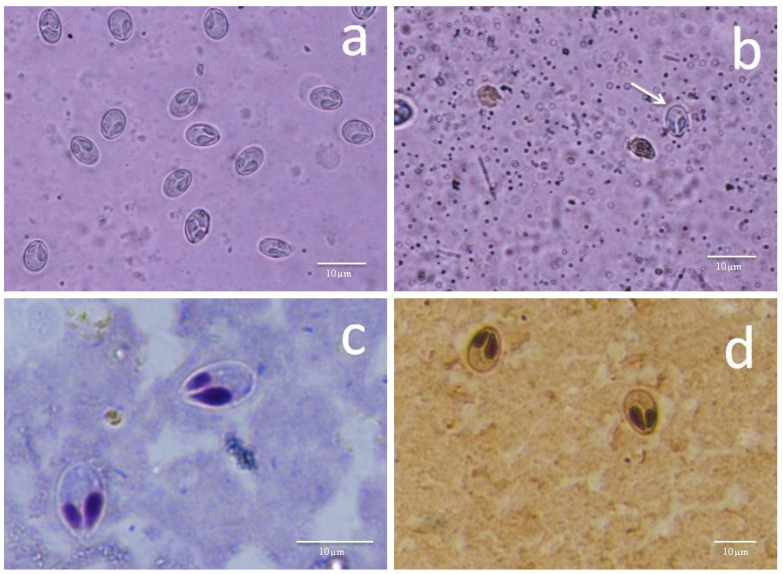
(i) Fresh myxospores of *M. grassi* sp. nov. from grass carp under phase contrast microscope. (**a**) Gill filament (**b**) liver (arrow); (ii) stained myxospores of *M. grassi* sp. nov. from the gill filament of grass carp. (**c**) giemsa stain (**d**) silver-nitrate stain.

**Figure 2:**
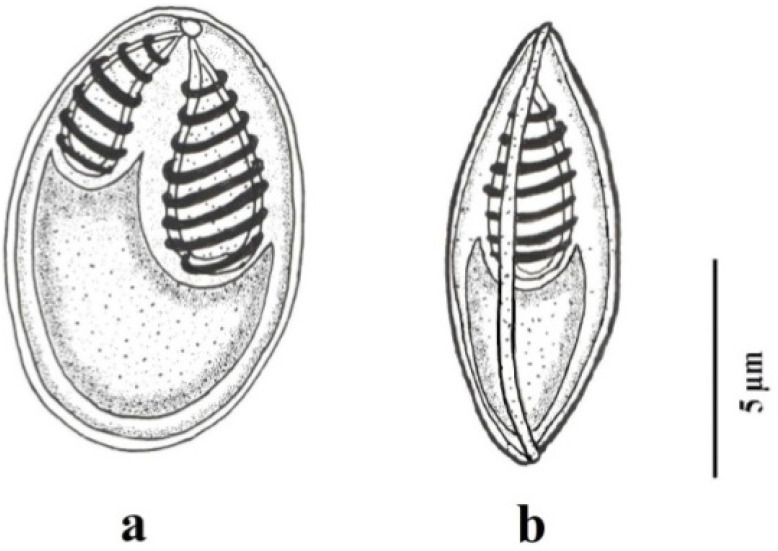
Camera Lucida drawings of the mature myxospore of *M. grassi* sp. nov. from the gill filaments of grass carp showing (**a**) frontal and (**b**) sutural view.

A total of 74 grass carps measured range 20–35 cm in length were examined, and gills and liver were infected in only 3 specimens (Table 1). However, less intensity of infection was observed in liver smears. Myxospores from both infected gills and liver were morphologically and morphometrically similar. Consequently, the infection in gills and liver were as primary and secondary site of infection respectively. Myxospores ellipsoidal, slightly pointed anteriorly (Figure 1 and Figure 2), measuring 6.0–11.1 (9.8 ± 0.9) μm long and 3.9–7.5 (6.2 ± 0.9) μm wide in valvular view (*n* = 34). Thickness of myxospore was 4.9–6.0 (5.4 ± 0.3) μm in sutural view. The ratio (LS/WS) of the myxospore was 1.58. The polar capsules were converging, drop-shaped and unequal. A larger polar capsule covers more than half of the myxospore body measuring 3.5–5.6 (4.6 ± 0.5) μm long and 1.5–2.6 (2.1 ± 0.2) μm wide. A smaller capsule length about 1/3rd of the myxospore length measured 2.8–4.5 (3.4 ± 0.4) μm long and 1.0–2.1 (1.5 ± 0.2) μm wide. The larger polar capsule consists of 8 coils while the smaller polar capsule consists of six coils in the polar filament. The range of coils is different according to the size of the polar capsule. The length of the polar filaments is 18.8–22.6 (20.3 ± 1.1) μm. The sporoplasm is homogenous, without iodinophilous vacuole.

### 2.2. Remarks on Morphological Analysis

First, it seems that *M. grassi* sp. nov. recorded from the grass carp during the present study resembled various *Myxobolus* species infecting gills and related organs. Besides that, species having ellipsoidal to pyriform myxospores and unequal polar capsules were compared with the present species *M. grassi* sp. nov. Particularly, the closely resembling species were being discussed here in detail: *Myxobolus diversus* and *Myxobolus bhadrensis* were relatively similar to the present species but *M. diversus* myxospores possessed a drop-shape with a pointed tip less wide than the present species while the sporoplasm of *M. bhadrensis* was coarse, uninucleated with extracapsular cavity and iodinophilous vacuole. *M. puntuisii* has smaller polar capsules whereas *M. naini* possessed larger myxospores and polar capsules. In addition, myxospores of *M. buccoroofus* from buccal cavity of *Labeo bata* were longer than the present species. *M. analfinus* myxospores from anal fin of *Heteropneustes fossilis* were bigger in size and has relatively smaller polar capsules while myxospores of *M. debsantus* from tail fin of *Catla catla* and *Labeo rohita* were wider than the present species. Myxospores of *M. burti* from muscles of *Notropis hudsonius* were longer and wider. Myxospores of *M. haldari* from fins and gills of *Cirrhinus mrigala* and *Labeo rohita* were also somewhat smaller and wider in comparison to the present species. *M. grassi* sp. nov myxospores were much wider than *M. intramusculi* from muscle cells of *Percopsis omiscomaycus*. *M. lalithae* from gill filaments of *Labeo calbasu* and *M. mathurii* from gills of *Puntius sarana* have also larger myxospores. Myxospores of *M. tripurensis* from gill filaments and gill rakers of *Labeo calbasu*; *L. bata* and *Cirrhinus reba* differed from *M. grassi* sp. nov. in the length of myxospore and in having sutural markings at the posterior end (Table 2).

### 2.3. Molecular Phylogeny

**Figure 3:**
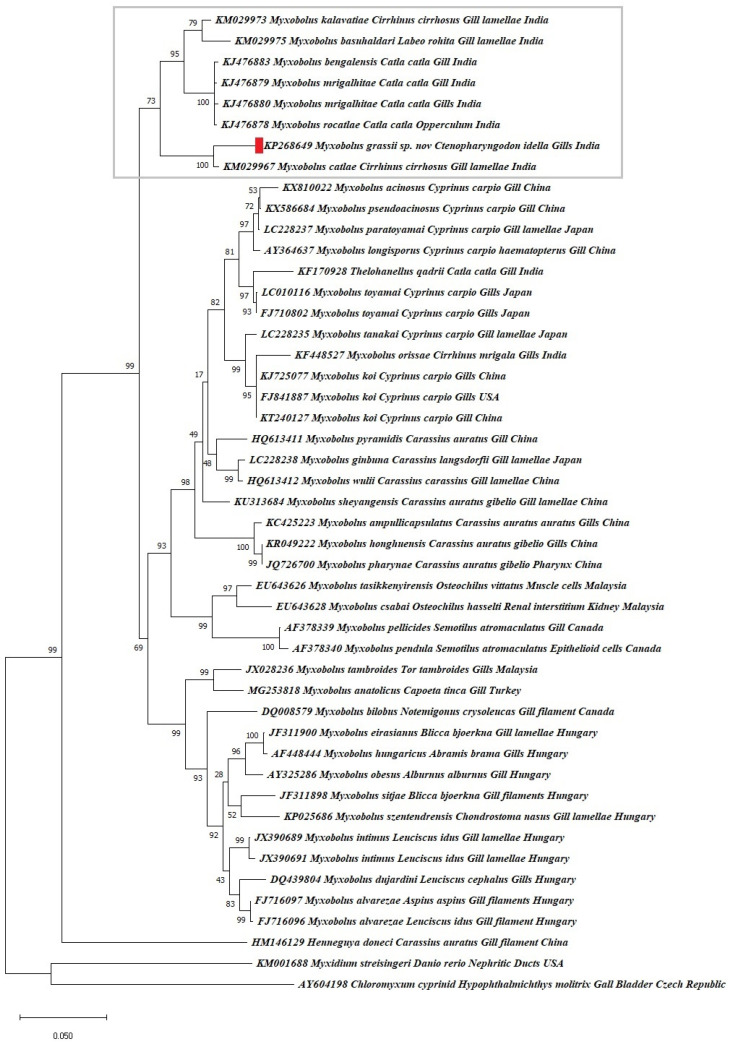
Phylogenetic tree constructed by maximum likelihood displaying *M. grassi* sp. nov. (KP268649) from *C.* *idella* clustering next to the Indian group of myxosporean. GenBank accession numbers are given before the species and numbers on the nodes represents the bootstrap confidence values.

In the support of morphological study, 970 bp fragment of 18S (SSU rDNA) gene of *M. grassi* sp. nov. was submitted to GenBank with the accession no. KP268649. Although only about half the target gene was amplified, it included coverage of conserved and variable regions. Additionally, there was a lack of intraspecific variation within the three isolates. The present partial sequence showed 98% homology with *M. catlae*, 93% homology to *M. kalavatiae* (Figure 3). In addition, other sequences with less similarity (90–92%) were used for phylogentic study. In phylogentic analysis, the present species was closely related to the gill isolated species *M. catlae* placing it to the same clade with high bootstrap value. Moreover, it also clustered giving the preference on the basal groups of sub clades with *Myxobolus* species infecting gills of Indian carps and numerous cyprinid fishes reported worldwide (Figure 3). This was further supported by the evolutionary divergence values of the present species with other related species. It was 0.03 with *M.* *catlae*, 0.06 with *M. kalavatae* and 0.20 with *Hennehuya doneci*, 0.26 with *Myxidium streisingeri* and 0.30 with *Chloromyxum cyprinid* (out group). Presumably, the association and connection within tissue-specific myxozoan groups is because of relationship after segregation and conversion into new species from old species. The rate of evolution analysis is usually a comparison where evolutionary change corresponds to a shift in the position of a species.

## 3. Discussion

The present species was compared with closely resembling species from gills and vital organs but that varied in morphologically. Furthermore, species with unequal, dimorphic myxospores from the synopses of Eiras et al. [6,7] have been compared with present species but all of the species were different in shape and size of myxospore. Moreover, the current species was compared with other Myxobolus species isolated from *C. idella* by earlier workers were as follows: *M. ctenopharyngodoni* from intestine, spleen and kidney; *M. huasaensis* from kidney; *M. microsporus* from almost all organs; *M. tricostatus* from gills and spleen; *M. pinna* from fins; and *M. edellae* from kidney. *M. ctenopharyngodoni*, *M. huasaensis*, *M. tricostatusis* and *M. edellae* all had comparatively different myxospores and the polar capsules were equal in size. *M. microspores* and *M. pinna* both have unequal polar capsules but were bigger in myxospore size.

The blast analysis of *M. grassi* sp. nov. exhibited 84–98% sequence similarity with as many as 28 myxobolid species infecting gills and related organs in India, Japan, Malaysia, Turkey, Canada, Hungary and Australia. A phylogentic study resulted in *M. grassi* sp. nov. being within the monophyletic clade comprising Indian species having 98% to 91% homology. On the other hand, other species such as *M. csabai* (90%), *M. pendula* (89%) and *M. pellicides* (89%) made a paraphyletic group of gill myxozoans in cyprinids, which indicates the relationship among host and aquatic environment i.e., freshwater fishes (Figure 3). According to Ferguson et al. [23], in the case of *Henneguya*, there is a strong tendency to form clades among species, based on the family of the host fish. The present species represents the Indian intracellular group exhibiting affinity to host family and geographical conditions while manifesting the basal groups of sub clades of *Myxobolus* species infecting intracellular-intramuscular group numerous cyprinid fishes reported worldwide i.e., it included myxosporeans species infecting other internal organs from Japan, Malaysia, Turkey, Canada and Hungary. Consequently, host tissue specificity is strict in myxosporeans, and still different habitats has been demonstrated for some species [24].

However, the difference of the resultant 18S rDNA sequence of *M. grassi* sp. nov. was again compared morphologically with the species assembling in monophyletic group, which indicated incongruence in morphological and molecular data. *M. grassi* sp. nov. clustering in sister clade with *M. catlae* having high bootstrap values differed morphologically and morphometrically with much elongated myxospore (approximately 2/3rd in size) and a sharply pointed thin tip having equal and longer polar capsules. It was well supported by the evolutionary divergence values of 0.03. Easy et al. [19] found 97.9% similarity between intracellular and intercellular plasmodia of *M. procerus*, parasite of *Percopsis omiscomaycus*, and designated a new species—*M.* *intramusculi* for the intracellular form. While other species clustered in the group of Indian cyprinids origin clade were also morphologically different with the present species. However, *M. kalavatiae* and *M. basuhaldari* were small, ovoid with equal polar capsules covering 1/3rd of the part of the myxospores while myxospores of *M. rocatlae* were elongated with sharply pointed anterior with slender long polar capsules. *M. mrigalhitae* possesses intercapsular process and iodinophilous vacuole whereas *M. bengalensis* comprises pear-shaped myxospores with longer, equal polar capsules and a spherical iodinophilous vacuole.

The present species infecting two organs i.e., gills and liver, can be considered as tissue-specific because maybe the species have developed somewhere else, as there were no developmental stages observed in both organs. Molnar et al. [25] stated site specificity of *M. shaharomae* from liver, testes and intestine was connected to the tissues rather than organs i.e., tissue specific, as the species develops plasmodia in blood vessels. The lower clade comprised of species from Hungary, Canada, Malaysia, Turkey and Australia with a very high bootstrap value indicating a close relationship and connection towards tissue and host tropism [26,27]. In view of close association involving histotropism and genetic distance among groups, the phylogenetic analysis leads us to consider *M. grassi* sp. nov. as a distinct new species after the comparison of the morphological, morphometric and molecular analysis.

## 4. Materials and Methods

### 4.1. Morphology and Morphometry

Seventy-four live specimens of grass carps were procured from Kaisar Bagh fish market (latitude 26°50′54.93″ N and longitude 80°55′52.08″ E), Lucknow, Uttar Pradesh, India. Fishes were transported live to the laboratory (in oxygenated plastic buckets and kept in aerated tank temporarily. Fishes were anesthetized with chloroform at a concentration of 40 mg/L and dissected after 2–3 days post capture. All the external and internal organs were screened for myxozoan infection. Organs were removed from freshly killed fish and placed in a Petri dish with saline, then microscopically examined under a stereo-zoom microscope SMZ-U (Nikon, Japan). Squash preparations of gills and liver were made and examined through a Nikon E600 microscope with 100X objective (plus immersion oil) for the presence of myxospores. Fresh smears revealed numerous myxospores (Figure 1a), however plasmodia were not detected. Fresh wet mount was treated with 10–12% KOH solution to evert the polar filaments. For permanent preparations, air-dried smears were stained with geimsa and silver nitrate [28] (Figure 1b). Fresh myxospores were photographed through a compound binocular microscope Eclipse E600 with Photomicrography digital camera DXM1200F (Nikon, Japan). Morphometric measurements of fresh myxospores (*n* = 30–34) were done with the help of software NIS-E-Br. Morphological characterization was carried out for the obtained parasite according to the guidelines of Lom and Arthur [29]. Morphometric data are presented in micrometers (μm) with ranges followed by mean ± SD in parentheses. Fresh myxospores from gill filaments (gill 2 and 3) (*n* = 20) and liver 1, 2, and 3 (*n* = 20) were measured.

### 4.2. Molecular Analysis

For molecular analysis, a gill sample from each of the three infected fish were taken. Morphometric measurements were obtained for the liver samples while contamination from host tissue prevented to obtain molecular data from liver. DNA extraction was performed from the myxospores (approximately 500–700) through DNeasy Blood & Tissue Kit (Qiagen, India) following the manufacturer’s protocol. DNA concentration was measured through Nano-drop 2000 spectrophotometer (Thermo Scientific, Waltham, MA, USA). Total 970 bp sequence was generated with MC5–MC3 [30] primer sets synthesized by Sigma-Aldrich, India and were used to amplify the partial 18S (SSU rDNA) gene by Polymerase Chain Reaction (PCR) in Eppendorf EP Gradient S Master Cycler (Eppendorf Inc., USA). The final volume of PCR 50 µL containing 1x PCR buffer, 0.2 mM dNTPs, 1.5 mM MgCl_2_, 0.25 μM of each primer, 2.0 U taq polymerase (Fermentas, Thermo Fisher Scientific) and 100 ng of the genomic DNA template [31]. The PCR amplification protocol consisted of initial denaturation at 94 °C for 4 min, followed by 35 cycles of denaturation at 94 °C for 50 s, at annealing of primers at 56 °C for 50 s, followed by 72 °C for 1 min, final extension at 72 °C for 7 min and then stored at 4 °C prior to sequencing. Aliquots (5 μL) of the amplicons were visualized with 0.02% bromophenol blue (G-Biosciences) after electrophoresis on 1% agarose gel. Amplicon was purified with the QIAquick Gel Extraction Kit (Qiagen), as per manufacturer‘s instructions. Purified amplicons were sequenced using the ABI BigDye Terminator Cycle Sequencing Ready Reaction Kit v3.1, using the ABI3730xl Genetic Analyzer (Applied Biosystems, Inc. Waltham, MA, USA) for the both directions. The alignment of nucleotide sequences was done with the help of Clustal W and MEGA X [32]. A query search for analogous nucleotide-nucleotide Basic Local Alignment Search Tool (blastn) [33] was conducted for comparison of the present sequence. The genetic distance in phylogenetic evaluation was computed by GTR + G model selected by analysis of best model for Maximum Likelihood (ML) (Figure 3) using MEGA X [31]. Bootstrap analysis (1500 pseudoreplicates) was employed to assess the evolutionary history of the taxa.

## 5. Conclusions

Grass carp have been reported from many countries and intensive culture practices makes this species susceptible to various parasitic diseases specifically myxozoans. Myxozoans are economically important groups of microscopic parasites among freshwater fishes especially in food and ornamental fishes. With this background, molecular characterization of the parasites is becoming increasingly important in order to identify the complex myxozoan parasites. The evolutionary assessment of present species shows the association of myxozoans species from fish host order and tissue specificity. Hence, details of the present myxospore based on morphological, morphometric and phylogenetic analysis suggest that *M. grassi* sp. nov. is a new species to the scientific world.

## Figures and Tables

**Table 1:** Comparative morphometric data of myxospores collected from gills and liver of 3 grass carp. (Gill 1 is the type sample for the study as having minimum and maximum range of morphometric measurements).

Tissue	Myxospore		Polar Capsules	
	Length (μm)	Width (μm)	Length (μm)	Width (μm)
Gill 1	6.0–11.1	3.9–7.5	3.5–5.6 (4.6 ± 0.5);	1.5–2.6 (2.1 ± 0.2);
	(9.8 ± 0.9)	(6.2 ± 0.9)	2.8–4.5 (3.4 ± 0.4)	1.0–2.1(1.5 ± 0.2)
Gill 2	7.0–10.7	4.0–7.1	3.5–5.3 (4.5 ± 0.5);	1.6–2.4 (2.0 ± 0.2);
	(9.7 ± 1.0)	(6.1 ± 0.9)	2.4–4.1 (3.5 ± 0.4)	1.0–2.0 (1.5 ± 0.2)
Gill 3	6.2–11.0	4.1–7.3	3.9–5.6 (4.7 ± 0.4);	1.5–2.5 (2.1 ± 0.2);
	(9.7 ± 1.2)	(6.2 ± 1.0)	3.0–4.1(3.5 ± 0.4)	1.2–1.8 (1.5 ± 0.1)
Liver 1	6.2–10.8	3.9–7.0	3.9–5.6 (4.6 ± 0.5);	1.5–2.4 (1.9 ± 0.2);
	(9.1 ± 1.5)	(5.9 ± 1.0)	3.0–4.3 (3.7 ± 0.4)	1.0–1.9 (1.4 ± 0.3)
Liver 2	7.0–10.9	4.0–7.0	3.8–5.2 (4.7 ± 0.4);	1.6–2.5 (2.1 ± 0.2);
	(9.5 ± 1.3)	(6.0 ± 0.9)	2.8–4.2 (3.5 ± 0.4)	1.0–1.8 (1.5 ± 0.2)
Liver 3	6.4–10.7	3.9–6.9	3.6–5.1 (4.6 ± 0.4);	1.5–2.4 (2.0 ± 0.2);
	(9.3 ± 1.1)	(6.0 ± 0.9)	2.8–4.3 (3.4 ± 0.4)	1.1–1.9 (1.5 ± 0.2)

**Table 2:** Comparative description of *M. grassi* sp. nov. with morphologically similar myxobolid species.

Species	Host	Site of Infection	Myxospore		Polar Capsule(s)		Locality	References
			Length (μm)	Width (μm)	Length (μm)	Width (μm)		
*M. grassi* sp. nov.	*C. idella*	Gills and liver	9.8 ± 0.9	6.2 ± 0.9	4.6 ± 0.5 (3.5–5.6);	2.1 ± 0.2 (1.5–2.6);	India	Present Study
			(6.0–11.1)	(3.9–7.5)	3.4 ± 0.4 (2.8–4.5)	1.5 ± 0.2 (1.0–2.1)		
*M. diversus*	*Schizothorax curvifrous*	Gill lamellae	9.1	5.8	4.1 (3.9–4.3);	1.6 (1.4–1.8);	India	[12]
			(8.9–9.3)	(5.3–6.4)	2.5 (2.2–2.8)	1.2 (1.0–1.4)		
*M. bhadrensis*	*Labeo rohita*	Muscle	9.5	7.14	3.5 (3.0–4.0);	2.2 (2.0–3.0);	India	[12]
			(8.0–11.0)	(7.0–8.0)	2.5 (2.0–4.0)	1.75 (1.0–2.0)		
*M. puntuisii*	*Puntius * *sophore*	Caudal fin	7.7	5.3	3.0 (2.9–3.0);	1.7 (1.6–1.8);	India	[13]
			(7.5–7.9)	(5.2–5.4)	1.8 (1.7–1.9)	0.9 (0.8–0.9)		
*M. naini*	*Labeo bata; * *L. rohita*	Gill filaments	15.1	9.2	6.3 (5.1–7.6);	2.9 (2.5–3.4);	India	[14]
			(13.6–16.6)	(7.9–10.5)	3.5 (3.1–3.9)	2.1 (2.0–2.2)		
*M. buccoroofus*	*Labeo bata*	Buccal cavity	11.6–12.7	6.4–8.1	4.5–5.3 (4.9);	2.7–3.0 (2.9);	India	[15]
			(12)	(7.1)	2.0–2.9 (2.5)	1.3–1.7 (1.5)		
*M. analfnus*	*Heteropneustes fossilis*	Anal fin	11.1–13.4	7.8–9.3	3.2–4.9 (4.1 ± 0.4);	2.0–2.4 (2.2 ± 0.1);	India	[16]
			(12.3 ± 0.7)	(8.6 ± 0.4)	2.0–3.1 (2.5 ± 0.3)	1.6–2.0 (1.8 ± 0.1)		
*M. burti*	*Notropis * *hudsonius*	Muscles	9.7–11.3	7.1–8.4	4.0–5.8 (5.3 ± 0.5);	2.1–3.2 (2.7 ± 0.3);	Canada	[17]
			(10.3 ± 0.6)	(7.7 ± 0.4)	4.3–5.2 (4.7 ± 0.3)	2.2–2.7 (2.5 ± 0.2)		
*M. debsantus*	*Catla catla; Labeo rohita*	Caudal fin	8.5–9.6	8.1–8.9	4.0–4.6 (4.3 ± 0.17);	2.0–2.6 (2.3 ± 0.18);	India	[16]
			(9.0 ± 0.2)	(8.4 ± 0.2)	2.6–2.9 (2.8 ± 0.09)	1.6–1.9 (1.8 ± 0.09)		
*M. haldari*	*Cirrhinus mrigala; * *Labeo rohita*	Fins and gills	9.0–10.0	7.0–8.5	4.0–5.0 (4.3);	2.5–3.0 (2.9);	India	[18]
			(9.3)		2.5–3.0 (2.9)	1.5–2.0 (1.9)		
*M. intramusculi*	*Percopsis * *omiscomaycus*	Muscle cells	9.9–15.7	4.6–8.0	4.0–7.9 (5.8 ± 0.6);	1.0–2.7 (1.7 ± 0.4)	Canada	[19]
			(12.5 ± 0.9)	(6.2 ± 0.6)	3.4–7.7 (5.8 ± 0.7)	0.9–0.7 (1.7 ± 0.3)		
*M. lalithae*	*Labeo calbasu*	Gill filaments	9.0–11.0	8.0–9.0	5.0–6.0 (5.8);	3.0–3.5 (3.0);	India	[20]
			(10)	(8.4)	4.0–5.0 (4.8)	2.5–3.0 (2.8)		
*M. mathurii*	*Puntius * *sarana*	Gills	8.7–23.5	5.1–10.1	2.7–11.9;	1.8–4.6;	India	[21]
					2.7–7.8	1.8–4.6		
*M. tripurensis*	*L. calbasu; * *L. bata; * *C. reba*	Gill filaments and gill rakers	8.1–9.4	6.5–7.0	4.0–4.5 (4.2 ± 0.18);	2.0–2.6 (2.3 ± 0.2);	India	[22]
			(8.9 ± 0.4)	(6.7 ± 0.19)	2.5–2.8 (2.6 ± 0.1)	1.6–1.9 (1.7 ± 0.1)		

## Data Availability

The data supporting the conclusions of this article are included within the article. The sequences have been submitted to the GenBank database under the accession number KP268649.

## Nomenclature Acts

The nomenclature act of present work was registered under ZooBank. Identifier for this publication is: http://zoobank.org/urn:lsid:zoobank.org:pub:9306AE87-D87A-47B6-A336-FE0F93A330D6 (accessed on 11 May 2018).
